# Supplementary figures and images for: Astrogliosis and neuroinflammation underlie scoliosis upon cilia dysfunction
Source: eLife. 2024 Oct 10;13:RP96831. doi: 10.7554/eLife.96831 (PMC11466456; doi:10.7554/eLife.96831)

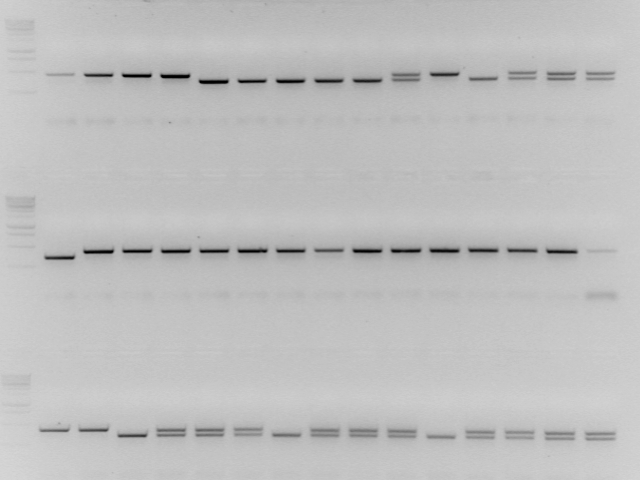

Supplement: Figure 1—figure supplement 1—source data 1. [file elife-96831-fig1-figsupp1-data1.tiff]

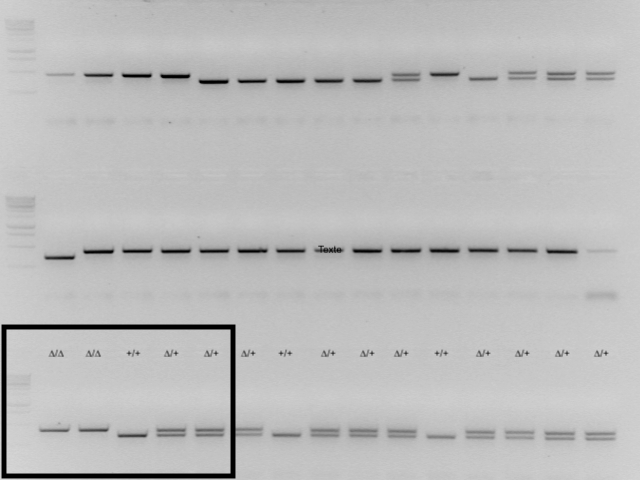

Supplement: Figure 1—figure supplement 1—source data 2. [file elife-96831-fig1-figsupp1-data2.tiff]

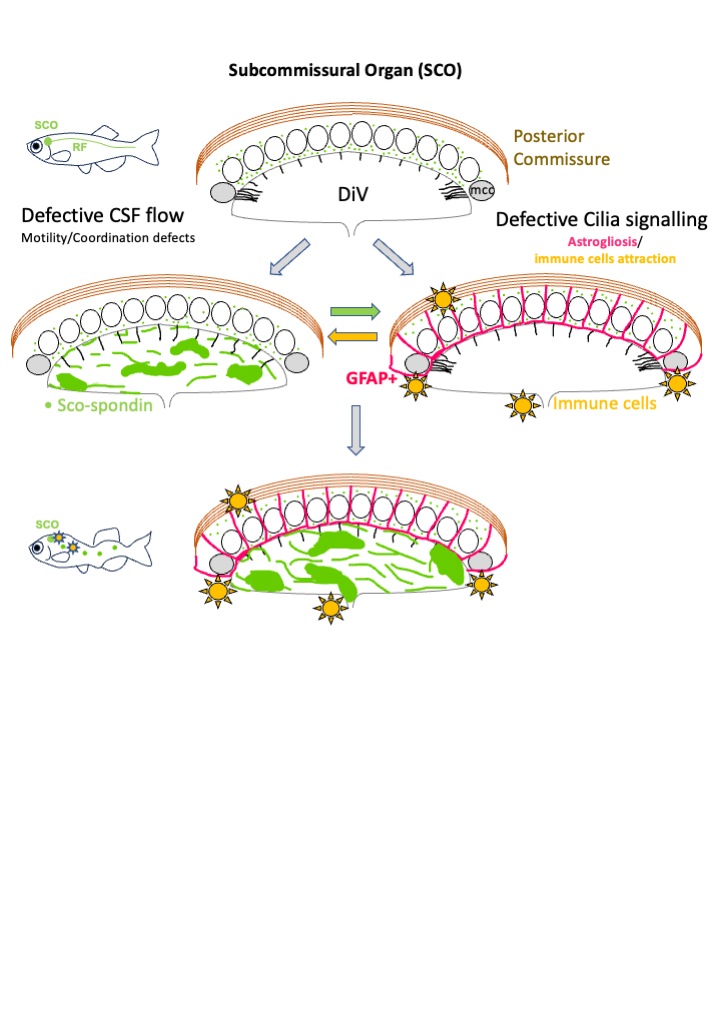

Supplement: Supplementary file 5. [file elife-96831-supp5.tiff]
